# Kinetic modeling of tumor growth and dissemination in the craniospinal axis: implications for craniospinal irradiation

**DOI:** 10.1186/1748-717X-1-48

**Published:** 2006-12-22

**Authors:** Jeffrey J Meyer, Lawrence B Marks, Edward C Halperin, John P Kirkpatrick

**Affiliations:** 1 Department of Radiation Oncology, Duke University Medical Center, Durham, NC, 27710, USA

## Abstract

**Background:**

Medulloblastoma and other types of tumors that gain access to the cerebrospinal fluid can spread throughout the craniospinal axis. The purpose of this study was to devise a simple multi-compartment kinetic model using established tumor cell growth and treatment sensitivity parameters to model the complications of this spread as well as the impact of treatment with craniospinal radiotherapy.

**Methods:**

A two-compartment mathematical model was constructed. Rate constants were derived from previously published work and the model used to predict outcomes for various clinical scenarios.

**Results:**

The model is simple and with the use of known and estimated clinical parameters is consistent with known clinical outcomes. Treatment outcomes are critically dependent upon the duration of the treatment break and the radiosensitivity of the tumor. Cross-plot analyses serve as an estimate of likelihood of cure as a function of these and other factors.

**Conclusion:**

The model accurately describes known clinical outcomes for patients with medulloblastoma. It can help guide treatment decisions for radiation oncologists treating patients with this disease. Incorporation of other treatment modalities, such as chemotherapy, that enhance radiation sensitivity and/or reduce tumor burden, are predicted to significantly increase the probability of cure.

## Background

Medulloblastoma is a relatively common primary tumor of the central nervous system (CNS) in the pediatric population, representing about 20% of brain tumors in this group [1]. The mainstays of treatment include maximal surgical resection followed by chemotherapy and radiation to the entire craniospinal axis (brain and spine), also known as craniospinal irradiation (CSI) [2]. Radiotherapists treat the entire craniospinal axis because the tumor cells have direct axis to the subarachnoid space, and, hence, the cerebrospinal fluid (CSF), which can provide a route for metastatic spread throughout the craniospinal axis. Early clinical studies indicated the importance of full CSI as opposed to treatment of smaller, gross-tumor-directed volumes [3]. Various clinical trials have been performed or are underway to study reduction of the radiation dose and attendant complications of CSI, possibly by way of intensifying chemotherapy. Nonetheless, CSI has retained its role as a critical component in the multimodality management of medulloblastoma [4,5].

Other primary and metastatic tumors of the CNS can also spread throughout the craniospinal axis via the CSF with leptomeningeal carcinomatosis, a descriptive term for tumor studding along the leptomeninges. In such patients, CSI may play a palliative role in the treatment armamentarium [6]. These patients are occasionally treated with intrathecal chemotherapy, which is another means of treating the entire subarachnoid space [7,8].

**Figure 1:**
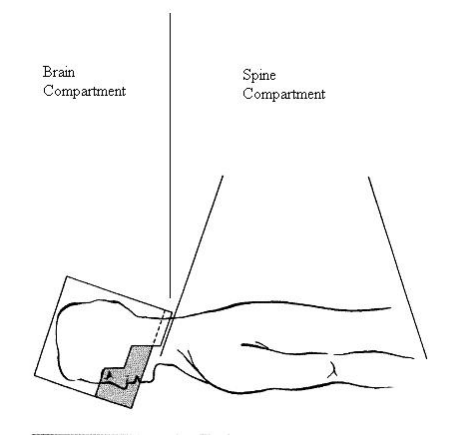
**Arrangement of craniospinal irradiation fields**. A lateral view of the relationship between a lateral portal and a posterior-anterior portal is shown. The location of the compartments is indicated. Within each compartment are two phases, namely the tumor and fluid phase. (Reproduced, with permission, with modifications, from L. E. Kun, Pediatric Radiation Oncology, eds. Edward C. Halperin, L. S. Constine, N. J. Tarbell, L. E. Kun, Lippincott Williams & Wilkins, 2005)

Delivery of CSI with standard photon therapy presents a geometric dilemma that is typically solved by the use of opposed lateral brain fields that are matched with collimator and treatment couch rotations to one or two posterior-anterior spine fields (Figure 1, reprinted with permission). When photons (as opposed to protons or electrons) are used to deliver CSI, these field arrangements ultimately lead to irradiation of a large portion of a patient's normal tissues, including the vertebral bodies with their productive bone marrow, as well as the viscera of the thorax, abdomen, and pelvis. Complications during treatment can include nausea, esophagitis, diarrhea and life-threatening myelosuppression (particularly in patients who have undergone preceding courses of chemotherapy); long-term complications may involve growth disturbances, hypothyroidism, and, especially in children, induction of second malignancies [9,10].

By the nature of their arrangement, the treatment fields described above functionally compartmentalize the craniospinal axis into 'brain' and 'spine' compartments. Because of acute treatment-related toxicities, especially myelosuppression (a complication that can arise early in the treatment course), it is occasionally necessary to suspend treatment of the spine temporarily while treatment of the brain continues. Since the brain and spine are in communication via the cerebrospinal fluid, holding treatment in one compartment may threaten tumor control in the other secondary to seeding of cells between these compartments. For example, tumor regrowth in the spine that occurs during treatment delays can seed tumor cells into the brain. CSF flow between the brain and spine may be considered analogous to the problem of a primary extracranial tumor forming distant metastases via hematogenous spread. Previous reports have modeled the process of metastasis, with the ultimate goal of evaluating and optimizing therapeutic intervention within the contexts of these models [11].

In this report we describe a kinetic model of tumor transport in the craniospinal axis (subarachnoid space and ventricle spaces) for medulloblastoma. The model is tested to assess if it can reasonably describe established clinical observations. Following this, the relative effects of changes in parameters incorporated in the model, such as those associated tumor cell shedding and adhesion, are discussed.

## Methods

**Figure 2:**
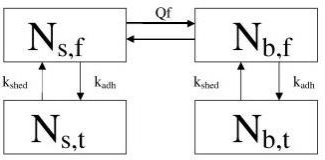
**The phases and compartments of the model**. The rate constants shown govern the flow of tumor between the phases.

The craniospinal axis is considered as having two tissue compartments, brain (b) and spine (s), with two phases, solid tumor (t) and cerebrospinal fluid (f), within each compartment (Figure 2). In the model the brain is not subdivided into supratentorial and posterior fossa (where medulloblastomas arise) compartments but rather as a single compartment. Recognizing that CSF flow is temporally and spatially heterogeneous [12], we assume that each fluid phase is well-mixed, as a crude approximation. *Between *the two compartments, cell transfer is governed by the volumetric flow rate, Q_f_, and the cell concentration in the fluid phases, i.e., the number of cells in the fluid phase divided by the volume of that phase. This is a reasonable assumption since the CSF flows relatively freely between the brain and spine compartments. *Within *each tissue compartment, transfer of cells between the phases is determined by the rate of adhesion of cells from the fluid phase onto the solid phase and by the rate of shedding of cells from the solid phase into the fluid phase. We assume that adhesion and shedding are described by the product of cell number and the rate constants k_adh _and k_shed_, respectively. However, not all of the cells shed into the fluid phase will be viable, and adhesion will account for only a portion of the cells cleared from the CSF. This is accounted for in the model by incorporating modulating efficiency factors for transfer of viable cells from the CSF to solid tumor and from solid tumor to CSF, γ_f _and γ_t_, respectively, which range in value from 0 to 1.

Finally, the tumor cell growth rate in each phase is assumed to be a linear function of tumor cell number (first-order growth kinetics), i.e., the product of growth rate constant, and cell number for that compartment and phase. For the purposes of this model, we are interested in estimating tumor control and focus on the development of relatively small tumors. Thus, we can ignore substrate and transport limitations that would require Gompertzian-type models of tumor growth [13]. Of course, much more complex growth models could be employed in this model, using the numerical solution technique described below.

Based on the above assumptions and in the absence of radiation-induced cell killing, the following system of ordinary differential equations is derived:

(1)    dN_s,f_/dt = k_g,f_N_s,f _+ Q_f_(N_b,f_/V_b _- N_s,f_/V_s_) + γ_t_k_shed_N_s,t _- k_adh_N_s,f_

(2)    dN_s,t_/dt = k_g,t_N_s,t _- k_shed_N_s,t _+ γ_f_k_adh_N_s,f_

(3)    dN_b,f_/dt = k_g,f_N_b,f _+ Q_f_(N_s,f_/V_s _- N_b,f_/V_b_) + γ_t_k_shed_N_b,t _- k_adh_N_b,f_

(4)    dN_b,t_/dt = k_g,t_N_b,t _- k_shed_N_b,t _+ γ_f_k_adh_N_b,f,_

where N_x,y _is the number of cells in compartment *x*, phase *y*; k_g,y _is the growth rate constant in phase *y*; and V_s _and V_b _are the volumes of the spine and brain subarachnoid space compartments, respectively. 's' refers to spine, 'b' refers to brain, 'f' refers to fluid, and 't' refers to tumor.

Rate constants in the model have been derived from *in vivo *data when possible so as to reflect clinical reality as closely as possible. Baseline values for these parameters are listed in Table 1. The value of k_g,t _used in the scenarios described in the results section (0.01 hr^-1^) is within the range of values that can be derived from the medulloblastoma potential doubling times (T_pot_) of 25 to 82 hours described in the work of Ito *et al *[14].

**Table 1:** Parameter values used in the base case

Parameter	Value	Units	Reference
k_g,t_	0.01	hr^-1^	14
k_g,f_	0.01	hr^-1^	14
D_s_	180	cGy	4
D_b_	180	cGy	4
D_0_	130	cGy	16
Q_F_	25	ml/hr	12
V_S_	25	ml	12, 15
V_B_	50	ml	12, 15
k_shed_	.001	hr^-1^	14
k_adh_	.0001	hr^-1^	14

The study by Ito *et al *also reported an observed clinical doubling time of 480–576 hours. Since there is, currently, no direct way of establishing k_shed_, we have estimated its value. By assuming that the discrepancy between T_pot _and observed doubling times is due solely to cells shedding from the tumor (and not from, for example, cell growth slowing with increasing tumor size nor from host immunologic attack of the tumor), we can establish an upper limit value for k_shed_; this value is close to 0.01 hr^-1^. Since this value for k_shed _has to be a gross overestimate (the other factors mentioned above do indeed contribute to the discrepancy between T_pot _and the observed doubling time), we have initially, arbitrarily, set it to a value that may be more in line with clinical reality, on the order of 0.001 hr^-1^. We have taken k_adh _to be 10% of the value of k_shed _(0.0001 hr^-1^), again as a rough estimate, with the assumption that it is more difficult for cells to adhere to other cells when they are flowing in the CSF. The values for k_shed _and k_adh _are both modulated by the values γ_f _and γ_t_, as described above.

The value for Q_f_, the volumetric flow rate and the spine and brain CSF volumes are taken from Bergsneider [12]. The values used for the volumes of the brain and spine CSF spaces are rough averages between what would be expected in a child and in an adult.

The system of equations can be discretized and re-arranged to yield the cell number at time *i+1 *as a function of the cell numbers at time *i*, yielding the following system of new equations:

(5)    N_s,f,i+1 _= N_s,f,i _+ Δt(k_g,f_N_s,f,i _+ Q_f_(N_b,f,i_/V_b _- N_s,f,i_/V_s_) + γ_t_k_shed_N_s,t,i _- k_adh_N_s,f,i_

(6)    N_s,t,i+1 _= N_s,t,i _+ Δt(k_g,t_N_s,t,i _-k_shed_N_s,t,i _+ γ_f_k_adh_N_s,f,i_)

(7)    N_b,f,i+1 _= N_b,f,i _+ Δt(k_g,f_N_b,f,i _+ Q_f_(N_s,f,i_/V_s _- N_b,f,i_/V_b_) + γ_t_k_shed_N_b,t,i _- k_adh_N_b,f,i_)

(8)    N_b,t,i+1 _= N_b,t,i _+ Δt(k_g,t_N_b,t,i _- k_shed_N_b,t,i _+ γ_f_k_adh_N_b,f,i_)

We then consider the situation in which a dose of radiation, D, is applied to a compartment over a short period of time, immediately prior to time *i+1*. We assume that D instantaneously reduces the number of cells capable of reproducing by a factor of e(D/D0)
 MathType@MTEF@5@5@+=feaafiart1ev1aaatCvAUfKttLearuWrP9MDH5MBPbIqV92AaeXatLxBI9gBaebbnrfifHhDYfgasaacH8akY=wiFfYdH8Gipec8Eeeu0xXdbba9frFj0=OqFfea0dXdd9vqai=hGuQ8kuc9pgc9s8qqaq=dirpe0xb9q8qiLsFr0=vr0=vr0dc8meaabaqaciaacaGaaeqabaqabeGadaaakeaacqqGLbqzdaahaaWcbeqaaiabcIcaOiabbseaejabc+caViabbseaenaaBaaameaacqaIWaamaeqaaSGaeiykaKcaaaaa@3406@. D_0 _is a parameter traditionally used to describe radiosensitivity and represents the dose required to reduce a clonogenic cell population to (ln 2)^-1^, or about 37%, of its initial value [16]. The D_0 _value ranged from 130 to 153 cGy for three cultured medulloblastoma cell lines studied *in vitro*, with a minimal shoulder to the curves as evidenced by the low extrapolation value of about 1.5 [17].

At time *i+1 *immediately following a dose of radiation, we can modify the above system of equations to yield:

(9)    N_s,f,i+1 _= e−Ds/D0
 MathType@MTEF@5@5@+=feaafiart1ev1aaatCvAUfKttLearuWrP9MDH5MBPbIqV92AaeXatLxBI9gBaebbnrfifHhDYfgasaacH8akY=wiFfYdH8Gipec8Eeeu0xXdbba9frFj0=OqFfea0dXdd9vqai=hGuQ8kuc9pgc9s8qqaq=dirpe0xb9q8qiLsFr0=vr0=vr0dc8meaabaqaciaacaGaaeqabaqabeGadaaakeaacqWGLbqzdaahaaWcbeqaaiabgkHiTiabdseaenaaBaaameaacqWGZbWCaeqaaSGaei4la8Iaemiraq0aaSbaaWqaaiabicdaWaqabaaaaaaa@34E3@ [N_s,f,i _+ Δt(k_g,f_N_s,f,i_+Q_f_(N_b,f,i_/V_b_-N_s,f,i_/V_s_)+ γ_t_k_shed_N_s,t,i_-k_adh_N_s,f,i_)]

(10)    N_s,t,i+1 _= e−Ds/D0
 MathType@MTEF@5@5@+=feaafiart1ev1aaatCvAUfKttLearuWrP9MDH5MBPbIqV92AaeXatLxBI9gBaebbnrfifHhDYfgasaacH8akY=wiFfYdH8Gipec8Eeeu0xXdbba9frFj0=OqFfea0dXdd9vqai=hGuQ8kuc9pgc9s8qqaq=dirpe0xb9q8qiLsFr0=vr0=vr0dc8meaabaqaciaacaGaaeqabaqabeGadaaakeaacqWGLbqzdaahaaWcbeqaaiabgkHiTiabdseaenaaBaaameaacqWGZbWCaeqaaSGaei4la8Iaemiraq0aaSbaaWqaaiabicdaWaqabaaaaaaa@34E3@ [N_s,t,i _+ Δt(k_g,t_N_s,t,i_-k_shed_N_s,t,i_+γ_f_k_adh_N_s,f,i_)]

(11)    N_b,f,i+1 _= e−Db/D0
 MathType@MTEF@5@5@+=feaafiart1ev1aaatCvAUfKttLearuWrP9MDH5MBPbIqV92AaeXatLxBI9gBaebbnrfifHhDYfgasaacH8akY=wiFfYdH8Gipec8Eeeu0xXdbba9frFj0=OqFfea0dXdd9vqai=hGuQ8kuc9pgc9s8qqaq=dirpe0xb9q8qiLsFr0=vr0=vr0dc8meaabaqaciaacaGaaeqabaqabeGadaaakeaacqWGLbqzdaahaaWcbeqaaiabgkHiTiabdseaenaaBaaameaacqWGIbGyaeqaaSGaei4la8Iaemiraq0aaSbaaWqaaiabicdaWaqabaaaaaaa@34C1@ [N_b,f,i _+ Δt(k_g,f_N_b,f,i_+Q_f_(N_s,f,i_/V_s_-N_b,f,i_/V_b_)+ γ_t_k_shed_N_b,t,i_-k_adh_N_b,f,i_)]

(12)    N_b,t,i+1 _= e−Db/D0
 MathType@MTEF@5@5@+=feaafiart1ev1aaatCvAUfKttLearuWrP9MDH5MBPbIqV92AaeXatLxBI9gBaebbnrfifHhDYfgasaacH8akY=wiFfYdH8Gipec8Eeeu0xXdbba9frFj0=OqFfea0dXdd9vqai=hGuQ8kuc9pgc9s8qqaq=dirpe0xb9q8qiLsFr0=vr0=vr0dc8meaabaqaciaacaGaaeqabaqabeGadaaakeaacqWGLbqzdaahaaWcbeqaaiabgkHiTiabdseaenaaBaaameaacqWGIbGyaeqaaSGaei4la8Iaemiraq0aaSbaaWqaaiabicdaWaqabaaaaaaa@34C1@ [N_b,t,i _+ Δt(k_g,t_N_b,t,i_-k_shed_N_b,t,i_+γ_f_k_adh_N_b,f,i_)]

where D_s _and D_b _are the doses administered in a single fraction to the spinal and brain compartments, respectively.

The equations were employed to numerically model various clinical scenarios, with adjustments made in different scenarios for the rate constants and for D_0_. Cell growth was not allowed in compartment *i *(i.e., k_g,i _was set to zero) if the number of cells N was less than 0.05, since it is at that point that the Poisson distribution, *e*^-N^, yields a tumor control probability of about 95%. Since we have not incorporated the effects of chemotherapy, a prescribed dose of 54 Gy to the brain and 36 Gy to the spine, administered at 1.8 Gy per day, has been used. This is the standard treatment regimen for a patient with medulloblastoma who is free from clinical evidence of disease outside the brain and negative CSF cytology [4]. Note that the model in its current formulation does not directly incorporate the effects of chemotherapy, which has emerged as a central component of therapy for patients with medulloblastoma. Chemotherapy may improve radisoensitvity, in addition to direct cytotoxic action on the tumor, improving outcome, as discussed below.

In all of the clinical scenarios, we have set N_b,t _to be 1 × 10^9 ^cells, roughly equal to the number of cells in one cm^3 ^of tumor, at t = 0. We have set N to be equal to 1, initially, in all other phases. Parameters for the initial set of scenarios are listed in Table 1.

## Results

### Scenario I

**Figure 3:**
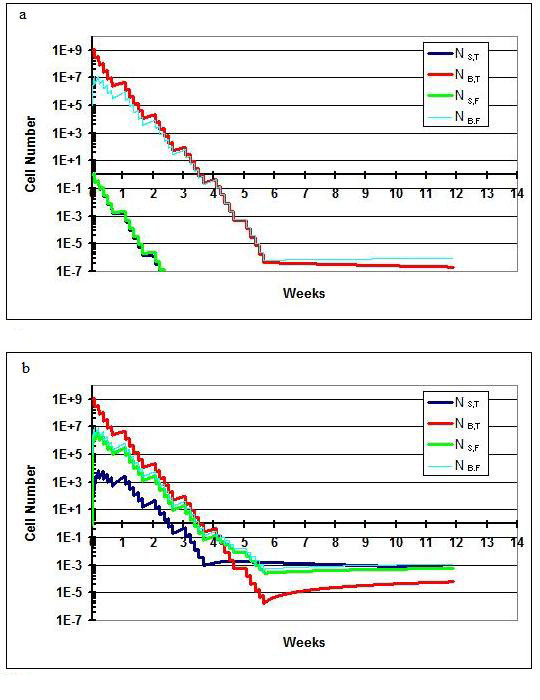
**Scenario I**. a) Treatment results when flow is not allowed between the brain and spine. The patient is cured. b) Treatment results when flow is allowed. The number of cells in the spine compartment quickly rises as a result of influx of cells from the brain compartment. The patient is nonetheless cured.

In this scenario (Figure 3), results following a standard course of treatment for the model allowing for flow (Q_f _= 25 ml/hr) and not allowing for flow (Q_f _= 0) are shown. Cure is achieved in both settings. This fits clinical experience; 54 Gy of radiation to the brain/posterior fossa and 36 Gy to the spine has a high probability of curing medulloblastoma. By adjusting various parameters such as k_g _and D_0_, it is obvious that differing outcomes would be observed. For example, if a patient's medulloblastoma cells were more radioresistant (i.e., had a higher D_0 _value), the outcome would not be as favorable. This is further discussed in scenario IV. Scenario I also shows that when flow between the spine and brain compartments is allowed there is a rapid rise in N_s,t _and N_s,f_.

### Scenario II

**Figure 4:**
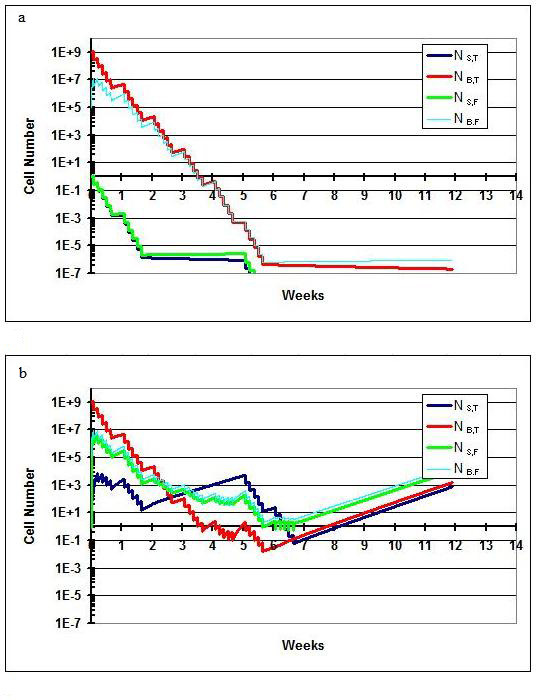
**Scenario II**. A break lasting three weeks is instituted. a) Treatment results when flow is not allowed. The number of cells in the spine compartment never reaches an appreciable level and the patient is cured. b) Tumor growth when flow is allowed between the brain and spine. The patient is not cured since the spine compartment is not sterilized.

In this scenario II (Figure 4), results following the introduction of a 3-week break in the spine portion of the treatment are described. As described above, such breaks may be necessitated when the acute reactions of the spine portion of CSI become life-threatening. The deleterious impact of treatment delay on outcomes in medulloblastoma has been documented in several retrospective series [18-20]. The kinetic model recapitulates this finding. In Figure 4a (with Q_f _= 25 ml/hr), the introduction of the break prevents sterilization of the spine phases, which were nearing sterilization just prior to the break. Enough cells remain to eventually repopulate all phases in the model. In a version of the model not allowing for flow (Q_f _= 0), shown in Figure 4b, the break never becomes an issue for cure because the spine is never seeded with cells from the brain. The brain compartment is easily sterilized with 54 Gy.

### Scenario III

**Figure 5:**
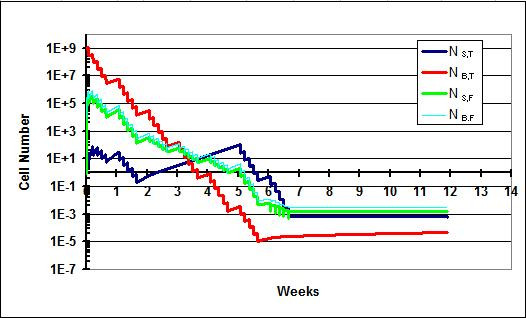
**Scenario III**. Treatment results when the value of k_adh _and k_shed _are lowered. Despite the treatment break of three weeks, cure is nonetheless achieved.

In this scenario (Figure 5), the importance of the parameter values in the model results is illustrated. Using the same scenario details as in scenario II, we have lowered the value for k_shed _and k_adh _by one order of magnitude each. This scenario models the response of tumors that are 'stickier' than those in the previous scenarios. Despite a three-week break, tumor control is nonetheless achieved. The reason is clear by comparison with Figure 5. By the time that the break is instituted, the value of N in the solid and fluid spine phases is significantly lower than in the previous scenario; seeding from the brain did not occur to the same extent since the cancer cells were less likely to be shed into the CSF.

### Scenario IV

**Figure 6:**
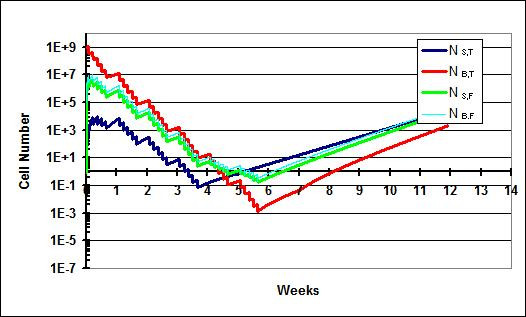
**Scenario IV**. Treatment results when the value of D_0 _is raised to 150 cGy. With greater tumor radioresistance, the patient is not cured.

In this scenario (Figure 6), the importance of the value of D_0 _is shown. We have used the original parameters as in scenario I, but increased the D_0 _value from 1.3 to 1.5 Gy. In this case, as a result of increased tumor radioresistance, cure is not achieved.

### The importance of the model parameters

**Figure 7:**
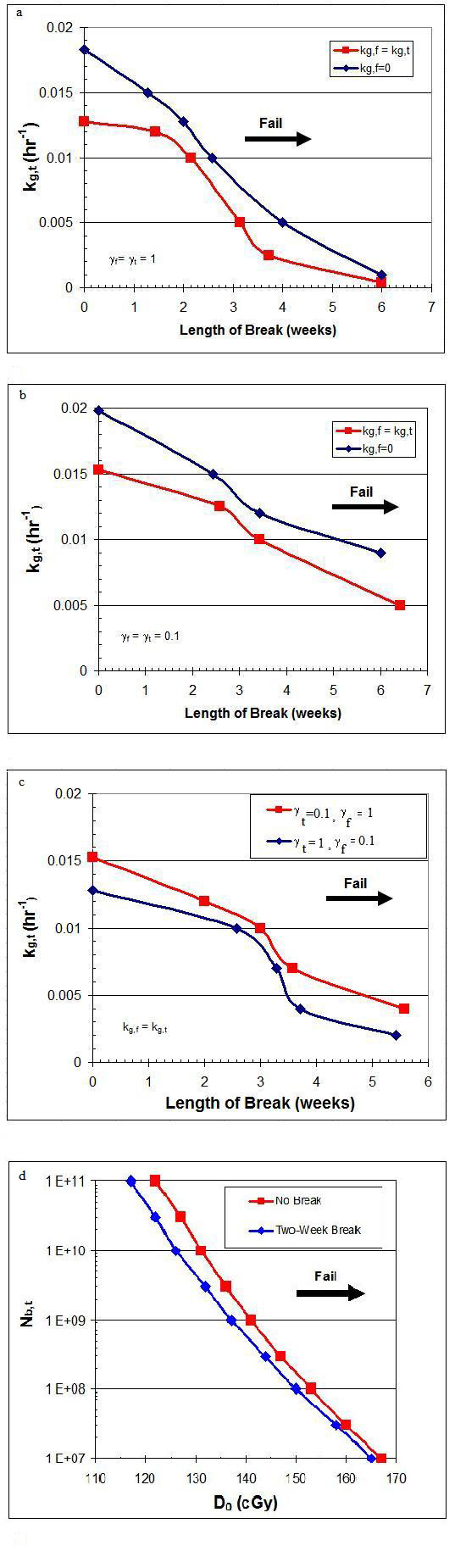
**Cross-Plot Analyses**. a) The interplay of k_g,t _and k_g,f _on treatment outcome, with and without growth of tumor cells in the fluid phase When k_g,f _is set to 0 (i.e., no growth of cells in the fluid phase), longer treatment breaks are allowed without threatening cure. b) The effect of efficiency factor γ is shown on treatment outcomes. Decreasing the efficiency of transfer of viable cells from one phase to the other (i.e., decreasing γ_t _and/or γ_f_) reduces the number of tumor cells, permitting a longer treatment break. c) The effect of independently varying γ_f _and γ_t _on treatment outcome is shown. High γ_t _and low γ_f _values versus the converse are associated with a higher risk of treatment failure for extended treatment breaks at all k_g _values. d) The effect of initial number of tumor cells in the brain parenchyma, N_b,t_, and radiosensitivity, D_0_, on treatment outcome is shown. Failure is more likely the higher the value of N_b,t _and D_0_.

It is clear from the above scenarios, as well as from clinical experience, that multiple factors likely determine if a course of therapy is curative or not for medulloblastoma. To illustrate the sensitivity of cure, cross-plot analyses of treatment outcome as a function of several tumor and transport parameters was undertaken. In Figure 7, the impact of the values of k_g,t_, k_g,f_, γ_f_, γ_t_, D_0 _and the initial size of the brain tumor (N_b,t_) on the maximum duration of treatment break duration is shown.

## Discussion

We have presented a two-compartment kinetic model that describes tumor growth and flow within the closed system of the craniospinal axis. Using model parameters derived from known experimental and clinical data, the simple model was able to generate results that are consistent with clinical observations. By such validation, it can be properly used by clinicians to achieve a 'first-approximation' prediction of various potential scenarios that may arise in the treatment of medulloblastoma.

The model and equations presented herein are a simplification of a complex process. Three major assumptions have been made in the model's creation. First is the assumption that the logarithm of cell survival is proportional to dose, or that the fraction of remaining cells is equal to e(-D/D0)
 MathType@MTEF@5@5@+=feaafiart1ev1aaatCvAUfKttLearuWrP9MDH5MBPbIqV92AaeXatLxBI9gBaebbnrfifHhDYfgasaacH8akY=wiFfYdH8Gipec8Eeeu0xXdbba9frFj0=OqFfea0dXdd9vqai=hGuQ8kuc9pgc9s8qqaq=dirpe0xb9q8qiLsFr0=vr0=vr0dc8meaabaqaciaacaGaaeqabaqabeGadaaakeaacqqGLbqzdaahaaWcbeqaaiabcIcaOiabb2caTiabbseaejabc+caViabbseaenaaBaaameaacqaIWaamaeqaaSGaeiykaKcaaaaa@34E7@. This is true for cells in the linear portion of cell survival curves, but not in the shoulder region where fractionated radiotherapy takes place. However, there is a minimal shoulder to medulloblastoma cell survival curves, so this assumption is probably reasonable [17].

Second, it has been assumed that the cells from the primary tumor are constantly disseminating in the CSF and forming satellite nodules that can then themselves disseminate immediately. This is almost certainly not the case for all tumors, especially those early in their growth [21].

Third is the fact that assumptions for the values of the rate constants have been made. The process of cell shedding from tumor masses in a circulating fluid, be it CSF or blood, is not well characterized, and the rate constants used in the analysis are extrapolations from limited data. The value of k_shed _and k_adh _are probably less than what was used in the analysis, since there are other factors besides cell shedding that make an observed doubling time for a tumor longer than T_pot_. It is also well known that not all tumors with access to the CSF circulate through it, or at least not to levels that lead to clinical complications, implying that k_shed _for these tumors is exceedingly low. For example, CSI was once the treatment of choice for intracranial germinomas [22,23]. However, more recent studies evaluating whole ventricle-only or whole brain-only treatment show that more limited treatment fields can lead to cure in many patients, indicating that (clinically relevant) spread to the spine is not a foregone conclusion in some diseases [24,25]. We have used the modulating factors γ_f _and γ_t _to describe the potential impact of changes in the k_shed _and k_adh _values on treatment outcome.

The assumption that there is no potential for 'escape' of cells circulating in the CSF to the circulatory system has also been made. This is a reasonable assumption given the exceeding rarity of extracranial metastases [4]. Many extracranial metastases are in fact intraperitoneal in origin, and arise in the setting of shunts that divert CSF into this space.

Finally, the assumption that the CSF contents are homogenous throughout the course of the craniospinal axis has been made. This may not be the case in all circumstances [26]. Incorporation of changes in cell density in the different compartments could be incorporated in future versions of the model. If tumor cell density is higher in the spine than in the brain, spine treatment breaks would likely lead to lower cure rates.

Why one tumor type can spread freely in the CSF and another remains more localized (i.e., why k_shed _and/or k_adh _differs between tumors) is not known. Molecular determinants of tumor cell invasiveness, such as cadherin expression, probably play a role. E-cadherin governs cell-cell contact and reduced expression of E-cadherin allows cells to separate from their neighbors and invade locally and distantly. Utsuki *et al *found E-cadherin was not expressed on any of the medulloblastoma cells studied [27]. Asano *et al *showed that reduced levels of N-cadherin were seen in astrocytic tumors that had disseminated via the CSF [28]. The values of k_shed _and k_adh _may in part be functions of the status of proteins such as the cadherins in tumors.

Although the growth rate constant for tumors used in the analysis is a reasonable value, the growth rate of cells circulating in the cerebrospinal fluid is less well understood. This environment may or may not be conducive to cell growth. Figure 7 shows the modest difference on treatment outcome between allowing versus not allowing tumor cell growth in the fluid phase of the model.

Despite these limitations, the model provides insight into the relationship between tumor growth, CSF flow, and radiation-induced cell killing. Modest changes in rate constant values, tumor growth rates, and/or tumor radiosensitivity will not change the general conclusions that emerge from it. Figure 7 again illustrates the potential impact of changes on certain of the model parameters on treatment outcome.

The cross-plots shown in Figure 7 may have direct clinical value for oncologists. Success or failure of a treatment regimen is quite sensitive to small variations in the starting tumor cell number and radiosensitivity. The most direct method of achieving a smaller initial tumor size is to perform a more complete surgery, though a maximum safe resection frequently dictates that some gross tumor be left behind to minimize morbidity. Alternatively, chemotherapy can reduce the tumor burden when administred before and/or with radiotherapy. In addition, chemotherapy may substantially increase radiosensitivity (i.e., decrease D_0_).

The parallels between CSF dissemination and hematogenous metastasis are obvious, but one point bears special mention. In our model, completion of the brain treatment initially leads to cure within this space (i.e., no tumor cells left). However, if the spine is left untreated, it will eventually re-seed the brain space and lead to tumor growth there. In this setting, the spine can be thought of as the 'primary' site and the brain as the 'metastatic' site. With the primary site left uncontrolled, the chance of developing metastatic sites is ultimately inevitable in this model. Many in the clinical oncology community have emphasized the importance of local therapies to prevent distant failures [29]. Aggressive attempts at local control can minimize such failures.

## Conclusion

Craniospinal irradiation remains an important component of the treatment of medulloblastoma. It is critical that clinicians are aware of the propensity of medulloblastoma cells to disseminate throughout the craniospinal axis. The model presented in this paper uses established medulloblastoma-related parameters to describe this dissemination and predict its complications. It reinforces the importance of good clinical practices, such as minimizing the duration of treatment breaks in the irradiation of the spinal fields, to improve the chance of favorable outcome. The model also suggests that the addition of other therapeutic modalities, such as chemotherapy, can significantly reduce the risk of treatment failure by relatively small improvements in radiosensitvity and/or lower tumor burden.

## Competing interests

The author(s) declare that they have no competing interests.

## Authors' contributions

JM helped conceive of the model, analyzed the scenarios, and drafted the manuscript. EH and LM provided insights into the model structure and edited the manuscript. JK conceived of the model and helped to draft the manuscript. All authors read and approved the final manuscript.
